# Effects of a gamma‐secretase inhibitor of notch signalling on transforming growth factor β1‐induced urethral fibrosis

**DOI:** 10.1111/jcmm.16837

**Published:** 2021-08-06

**Authors:** Shanlong Huang, Delai Fu, Ziyan Wan, Min Li, Hecheng Li, Tie Chong

**Affiliations:** ^1^ Department of Urology The Second Affiliated Hospital of Xi'an Jiaotong University Xi'an China; ^2^ Department of Pediatrics The Second Affiliated Hospital of Xi'an Jiaotong University Xi'an China

**Keywords:** DAPT, human urethral scar fibroblast, Notch signalling, transforming growth factor β1, urethral fibrosis, urethral stricture

## Abstract

Urethral stricture (US) is a common disorder of the lower urinary tract in men caused by fibrosis. The recurrence rate of US is high; however, there are no effective therapies to prevent or treat urethral fibrosis. The pathogenesis of urethral fibrosis involves myofibroblast activation and excessive extracellular matrix (ECM) deposition. The molecular mechanisms underlying this pathological activation are not completely understood. It has been demonstrated that Notch signalling contributes to the development of fibrosis and inflammation. However, whether this contributes to urethral fibrosis remains unclear. In this study, activation of Notch signalling was observed in patients with US. Additionally, it was noted that activation of Notch signalling promoted ECM production and myofibroblast activation in human urethral scar fibroblasts (HUSFs) treated with transforming growth factor (TGF) β1. However, the Notch inhibitor *N*‐[*N*‐(3,5‐difluorophenacetyl)‐L‐alanyl]‐S‐phenylglycine t‐butyl ester (DAPT) suppressed activation of Notch signalling as well as proliferation and migration of the TGFβ1‐treated HUSFs. Additionally, DAPT ameliorated TGFβ1‐induced urethral fibrosis in Sprague Dawley rats by suppressing ECM production, myofibroblast activation and the TGFβ signalling pathway. These findings demonstrate that Notch signalling may be a promising and potential target in the prevention or treatment of urethral fibrosis.

## 1 INTRODUCTION

Urethral stricture (US) is a common disorder in men characterized by narrowing of the urethra, which is caused by urethral and corpus spongiosum fibrosis.^1^ Complications of US include recurrent urinary tract infections and urinary retention. Currently, surgical strategies that are considered for US patients include urethral dilatation, urethrotomy and urethroplasty. However, stricture recurrence remains a challenge regardless of treatment.^2^, ^3^ Pathologically, lesions of the urethral tissue are chronically inflamed, and there is activation and accumulation of myofibroblasts, overexpression of collagen and excessive deposition of extracellular matrix (ECM), which lead to urethral fibrosis, and finally US.^4^, ^5^ Antifibrotic drugs are potentially used to enrich US treatment. However, the cellular and molecular mechanisms leading to urethral fibrosis remain unclear. Therefore, a better understanding of the mechanisms is crucial for exploring novel and precise pharmacological treatments to prevent urethral fibrosis.

Notch signalling, which is highly conserved in almost all animal species, is essential for regulating cell proliferation, differentiation and apoptosis.^6^ Currently, four Notch receptors (Notch1‐4) and two families of Notch ligands (Jagged and Delta‐like) have been described.^7^ Following activation of Notch signalling by binding of Notch ligands, Notch receptors are cleaved by the γ‐secretase complex with subsequent release of the Notch intracellular domain (NICD).^8^ Furthermore, translocation of NICD into the nucleus induces transcription of Notch signalling target genes such as Hes1.^9^ This signalling activation can be inhibited by the γ‐secretase inhibitor *N*‐[*N*‐(3,5‐difluorophenacetyl)‐L‐alanyl]‐S‐phenylglycine t‐butyl ester (DAPT).^10^ Abnormal activation of Notch signalling is involved in the pathogenesis of some human diseases, such as acute leukaemia, melanoma and systemic sclerosis.^11^, ^12^ Recent studies have demonstrated that Notch signalling contributes to the development of fibrotic diseases, including tissue fibrosis (pulmonary, liver, kidney and cardiac fibrosis).^13^

Transforming growth factor β1 (TGFβ1) plays a crucial role in the pathogenesis of fibrosis. Accumulating evidence indicates a vital role of TGFβ1 in the pathophysiology of urethral fibrosis.^14^, ^15^ Moreover, TGFβ can stimulate the Notch ligand Jagged1 and further activate Notch signalling through mothers against decapentaplegic homolog 3 (Smad3)‐dependent mechanisms.^16^ Additionally, Notch signalling regulates TGFβ‐induced cell migration and cell growth arrest in epithelial cells.^17^ These mechanistic studies support crosstalk or interactions between TGFβ and Notch signalling. US involves fibrosis, and however, it is not clear whether Notch signalling promotes urethral fibrosis. Therefore, inhibition of Notch signalling may be a promising and potential therapeutic target in the management of urethral fibrosis.

In the present study, we investigated the potential role of Notch signalling in urethral fibrosis development and whether suppression of Notch signalling activation by DAPT can alleviate urethral fibrosis in vitro and in vivo. For the first time, we demonstrated that Notch signalling is activated in urethral fibrosis and plays an important role in ECM production and myofibroblast activation.

## 2 MATERIALS AND METHODS

### 2.1 Ethics statement

The protocols for the human and animal studies were approved by the Ethics Committee of Xi'an Jiaotong University (Xi'an, China). Informed consent and approval were obtained from all participants. The study of human tissues was performed according to the guidelines of the Research Committee of Xi'an Jiaotong University and the Declaration of Helsinki.

### 2.2 Clinical tissues and primary cell culture

Normal and scar urethral tissues were obtained from six male patients with US undergoing primary urethroplasty at The Second Affiliated Hospital of Xi'an Jiaotong University (Xi'an, China). Normal urethral tissues were collected from the same patients (Table 1). None of the patients had tumours, infectious diseases or autoimmune diseases. The locations of the strictures were all at the membranous urethra. Clinical samples were collected following surgery and stored at −80°C for further protein and RNA extraction.

**TABLE 1:** Clinical features of patients with urethral stricture

Number	Age	Sex	Location	Length	Cause
1	45	Male	Membrane	1.5 cm	Traumatic
2	53	Male	Membrane	0.8 cm	Traumatic
3	57	Male	Membrane	1.2 cm	Traumatic
4	62	Male	Membrane	1.3 cm	Traumatic
5	58	Male	Membrane	1.2 cm	Traumatic
6	65	Male	Membrane	1.5 cm	Traumatic

Primary human urethral scar fibroblasts (HUSFs) were established and cultured as previously described.^18^ Briefly, isolated samples were pre‐treated with collagenase (3 ml, 30 mg/ml; Sigma‐Aldrich) and seeded in Dulbecco's modified Eagle's medium (Invitrogen) supplemented with 20% foetal bovine serum (Gibco) and 1% penicillin‐streptomycin at 37°C with 5% CO_2_. The cells surrounding the explants were passaged. HUSFs from passages 4–8 were used in the experiments.

### 2.3 Immunofluorescence study

Human urethral scar fibroblasts were fixed in 4% paraformaldehyde and permeabilized with 0.5% Triton. The fixed cells were then incubated with primary antibodies against vimentin (1:200) and alpha smooth muscle actin (α‐SMA, 1:200) (Cell Signaling Technology) primary antibodies overnight at 4°C. Fluorescein isothiocyanate‐conjugated goat anti‐rabbit and Alexa Fluor‐conjugated goat antibodies (Invitrogen) were used as secondary antibodies. Fluorescence confocal images were captured using a Zeiss Observer I fluorescence microscope (Zeiss).

### 2.4 Cell growth assay

Human urethral scar fibroblasts were seeded in 96‐well plates, after which their viability was measured in a WST‐8 assay using a Cell Counting Kit‐8 (CCK‐8; Dojindo). Briefly, cells were treated with varying concentrations of DAPT for 24, 48 or 72 h with or without TGFβ1. CCK‐8 solution (10 μl) was added to each well, and the plates were incubated at 37°C for 2 h. Absorbance was measured at 450 nm.

### 2.5 Cell migration assay

Pre‐treated HUSFs were seeded in the upper chambers of 8 μM pore size inserts (BD Biosciences). The lower chambers were filled with complete medium containing 10% foetal bovine serum. The cells were incubated at 37°C for 24 h, and then, the inserts were fixed with methanol and stained with 0.1% crystal violet (Sigma‐Aldrich). The migrated cells were captured using a microscope (Leica DM IL inverted microscope, 10×) and analysed using ImageJ software (National Institutes of Health).

### 2.6 Coimmunoprecipitation assay

Human urethral scar fibroblasts were starved in serum‐free medium for 4 h and then treated with TGFβ1 (10 ng/ml) for 1 h. Triton X‐100 cell lysates were immunoprecipitated with anti‐NICD antibodies that were covalently coupled to Sepharose beads (Amersham Biosciences). The beads were then incubated at 4°C for 2 h. Immunoblots were probed with anti‐Smad3 antibody (Cell Signaling).

### 2.7 Cell transfection

Human urethral scar fibroblasts were transfected with lentiviral constructs expressing NICD at multiplicity of infection of 50 (GenePharma) according to the manufacturer's instructions. Overexpression efficiency was evaluated using green fluorescent protein‐positive cells under a fluorescence microscope and by Western blotting.

### 2.8 Rat model of urethral fibrosis and treatment

Adult male Sprague Dawley rats (*n* = 24, 300–350 g) (Centre of Laboratory Animals, The Medical College of Xi'an Jiaotong University, Xi'an, China) were randomly divided into control (saline, *n* = 8), TGFβ1 (*n* = 8) and TGFβ1 + DAPT (*n* = 8) groups for the study. All the treatments were administered as injections into the urethra of the rats. Urethral fibrosis was induced in rats in the TGFβ1 and TGFβ1 + DAPT groups with TGFβ1 local injection as described previously.^15^ Briefly, all the rats were anaesthetized with intravenous pentobarbital (30 mg/kg) and fixed in the supine position. The ventral penile skin was then incised to visualize the urethra. Next, 10 µg TGFβ1 was injected into the urethral wall at the 3, 6, 9 and 12 o'clock positions with a 30‐gauge needle. Rats in the control group were injected with saline. After 24 h, all the rats were continuously administered a second injection at the same position with either saline (control and TGFβ1 groups) or 1 mg DAPT (TGFβ1 + DAPT group). One month later, urethral tissues were harvested for the evaluation of urethral fibrosis.

### 2.9 Western blot analysis

Total proteins were extracted from clinical samples, HUSFs and rat urethral tissues for analysis. The isolated proteins (20 µg) were separated by 10% sodium dodecyl sulphate‐polyacrylamide gel electrophoresis, transferred onto polyvinylidene difluoride membranes (Millipore) and probed overnight at 4°C with primary antibodies specific to Notch1 (1:1000), Notch2 (1:1000), Notch3 (1:1000), Notch4 (1:1000), Jagged1 (1:1000), Jagged2 (1:1000), Delta1 (1:1000), Delta3 (1:1000), Delta4 (1:1000), Hes1 (1:1000), P‐Smad3 (1:1000), Smad3 (1:1000) (Cell Signaling Technology), NICD (1:1000), collagen I (1:1000), collagen III (1:1000), fibronectin (1:1000) and α‐SMA (1:1000) (Abcam). The membranes were then incubated with a secondary antibody (horseradish peroxidase‐conjugated goat anti‐mouse or anti‐rabbit IgG antibody) (ZSGB‐BIO) and imaged by enhanced chemiluminescence (Amersham). Glyceraldehyde 3‐phosphate dehydrogenase was used as a protein‐loading control.

### 2.10 Quantitative reverse transcription polymerase chain reaction (RT‐qPCR)

Total RNA from clinical samples, HUSFs and rat urethral tissues was extracted using an RNA isolation kit (Takara Biochemical) or TRIzol Reagent (Thermo Fisher Scientific) according to the manufacturer's instructions. A total of 2 µg of RNA was reverse transcribed using PrimeScript RT reagent kit (Takara Biochemical). RT‐qPCR was performed using a Bio‐Rad iQ5 PCR system (Bio‐Rad). The primer sequences for each gene are listed in Table 2.

**TABLE 2:** Primer sequence

	Genes	Forward primers	Reverse primers
Human	Notch1	GGACCTCATCAACTCACA	GTCTCCTCCCTGTTGTTC
	Notch2	GGTCTCAGTGGATATAAGTG	CTGGCATGGATTCGAAAG
	Notch3	CGGCTAAAGGTAGAGGAG	CAACCAGATGGTGTTGAG
	Notch4	GCATTGGTCTCAAGGCAC	CCTGTTTCTTCAGCCTGG
	JAG1	GATGTCACCAGGTCTTACTAC	GTATATCTTCAGCAGAAATGG
	JAG2	CACTGCTCCTGGCTGTCAC	AGGCACCACACAGCACAG
	DLL1	GGTGGAGAAGCATCTGAA	CTTCCATTTTACACCTCAGTTG
	DLL3	CAGCTGTAGTGAGACACC	GCAGATGTAGGCAGAGTC
	DLL4	GCGGTTACACAGTGAAAA	CTCCTGCCTTATACCTCC
	Hes1	GCACAGAAAGTCATCAAA	GTGCTTCACTGTCATTTC
	COL1A1	CCCTCCCCAGCCACAAAGAGTCT	GGGTGACTCTGAGCCGTCGG
	COL3A1	GGGAATGGAGCAAGACAGTCTT	TGCGATATCTATGATGGGTAGTCTCA
	ACTA2	ACTGGGACGACATGGAAAAG	TACATGGCTGGGACATTGAA
	FN1	CCAACCTACGGATGACTCGT	GCTCATCATCTGGCCATTTT
	GAPDH	TGGCGCTGAGTACGTCGTG	ATGGCATGGACTGTGGTCAT
Rat	COL1A1	TGAACGTGACCAAAAACCAA	AAGGAACAGAAAAGGCAGCA
	COL3A1	GTCCACGAGGTGACAAAGGT	CATCTTTTCCAGGAGGTCCA
	ACTA2	ACTGGGACGACATGGAAAAG	CATCTCCAGAGTCCAGCACA
	FN1	GAAAGGCAACCAGCAGAGTC	CTGGAGTCAAGCCAGACACA
	GAPDH	AGACAGCCGCATCTTCTTGT	CTTGCCGTGGGTAGAGTCAT

### 2.11 Histologic analysis

The harvested rat urethral tissues were fixed in 4% paraformaldehyde solution and processed using routine methods. Cross sections of the urethral tissues were stained with Masson's trichrome (MT) and haematoxylin and eosin (H&E) as previously described.^19^ Representative images were acquired using the CaseViewer software.

### 2.12 Statistical analysis

All the data have been presented as mean ± standard error of the mean (SEM). A minimum of three independent replicates of each experiment were performed. GraphPad Prism 7.0 (GraphPad Software) was used for statistical analysis. Multiple groups were assessed using one‐way analysis of variance. Differences in data were considered statistically significant at *p* < 0.05.

## 3 RESULTS

### 3.1 Notch signalling is activated in the scar tissues of US patients

**FIGURE 1:**
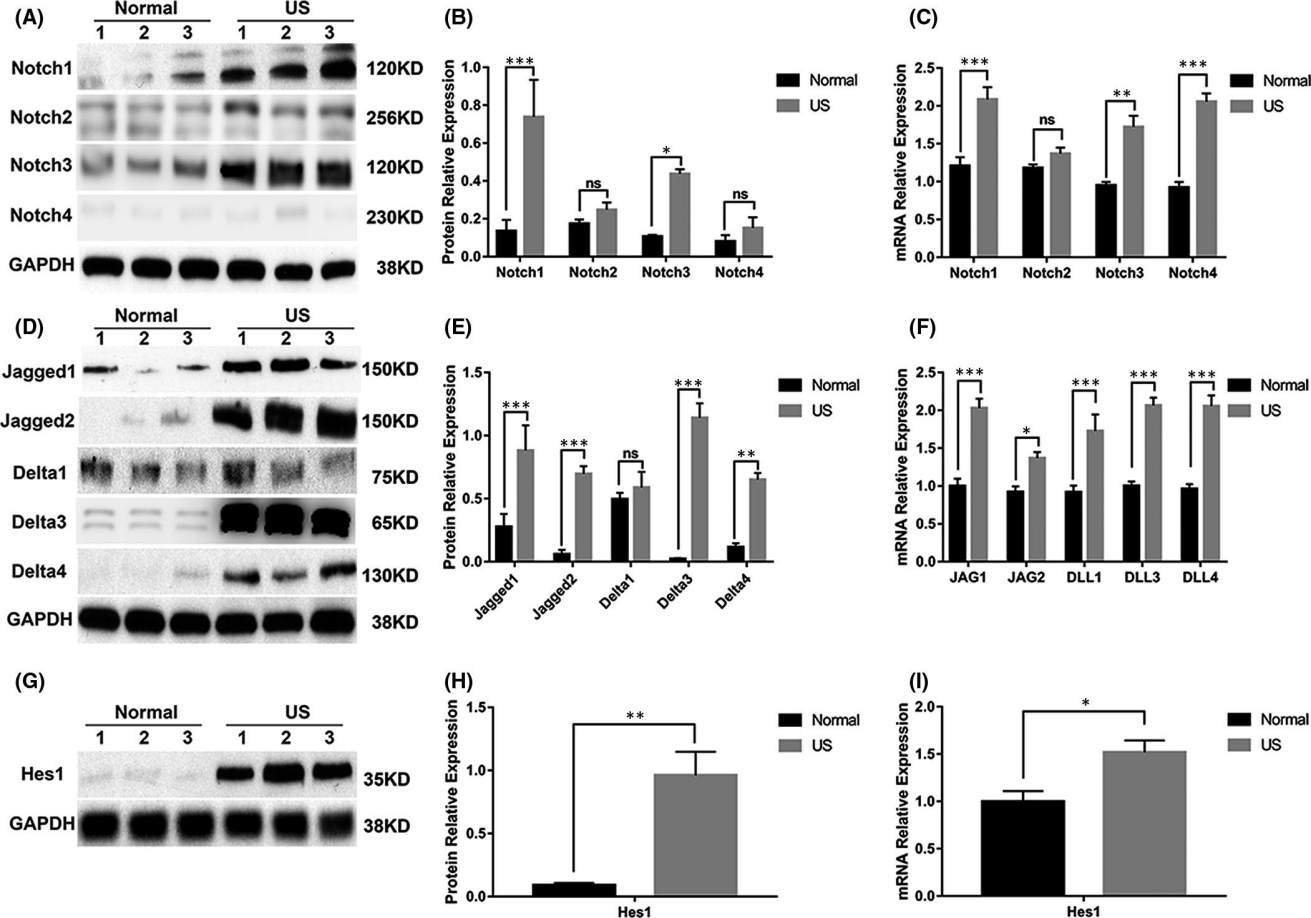
Notch signalling is activated in the scar tissues of patients with US. (A) Western blot analysis of Notch receptors. Expression of Notch1‐4 in normal urethra (normal group) and tissues isolated from three patients with US. (B) Quantification of the relative expression of Notch1‐4 proteins. (C) Real‐time PCR analysis of Notch receptors. Relative expression of Notch1‐4 mRNA in the normal and US groups. (D) Western blot analysis of Notch ligands. Jagged1, Jagged2, Delta1, Delta3 and Delta4 expression in the normal and US groups. (E) Quantification of the relative expression of Jagged1, Jagged2, Delta1, Delta3 and Delta4 proteins. (F) Real‐time PCR analysis of Notch ligands. Relative expression of JAG1, JAG2, DLL1, DLL3 and DLL4 mRNA in the normal and US groups. (G) Western blot analysis of the Notch target gene Hes1 in the normal and US groups. (H) Quantification of the relative expression of Hes1 proteins. I, Real‐time PCR analysis of Hes1. Relative expression of Hes1 mRNA in the normal and US groups. Data are expressed as mean ± SEM (*n* = 3, normal group; *n* = 3, US group). * indicates *p* < 0.05, ** indicates *p* < 0.01, and *** indicates *p* < 0.001. ns: no significant difference

To determine whether Notch signalling is involved in scar tissue formation in US patients, we first analysed the expression of the four Notch receptors in the patients. Western blot analysis and quantification revealed that the protein levels of Notch1 and Notch3 were significantly higher in the US group than in the normal group (Figure 1A,B). Although Notch2 was detectable, its protein levels did not differ between the US and normal groups (Figure 1A,B). In contrast to Notch1, 2 and 3, Notch4 was barely detectable in either group (Figure 1A,B). Furthermore, real‐time PCR analysis showed that the mRNA levels of Notch1, 3 and 4 were significantly upregulated in the US group compared to their respective levels in the normal group (Figure 1C). In contrast, the levels of Notch2 mRNA did not differ between the two groups.

The expression of Notch ligands and Notch target genes were also analysed to investigate the mechanisms that activate Notch signalling in US patients. We observed a significant expression of Notch ligands such as Jagged1, Jagged2, Delta3 and Delta4, except Delta1, in the US group compared to the normal group (Figure 1D,E). Consistently, the mRNA levels of Jagged1, Jagged2, Delta1, Delta3 and Delta4 were upregulated in the US group (Figure 1F). A prominent expression of the Notch target gene Hes1 was observed in the US groups (Figure 1G,H). Consistent with the results at the protein level, the levels of Hes1 mRNA were upregulated in the US group compared to those in the normal group (Figure 1I). These results suggest that Notch signalling is activated in US scar tissue.

### 3.2 Notch inhibitor DAPT suppresses activation of Notch signalling as well as proliferation and migration of TGFβ1‐treated HUSFs

**FIGURE 2:**
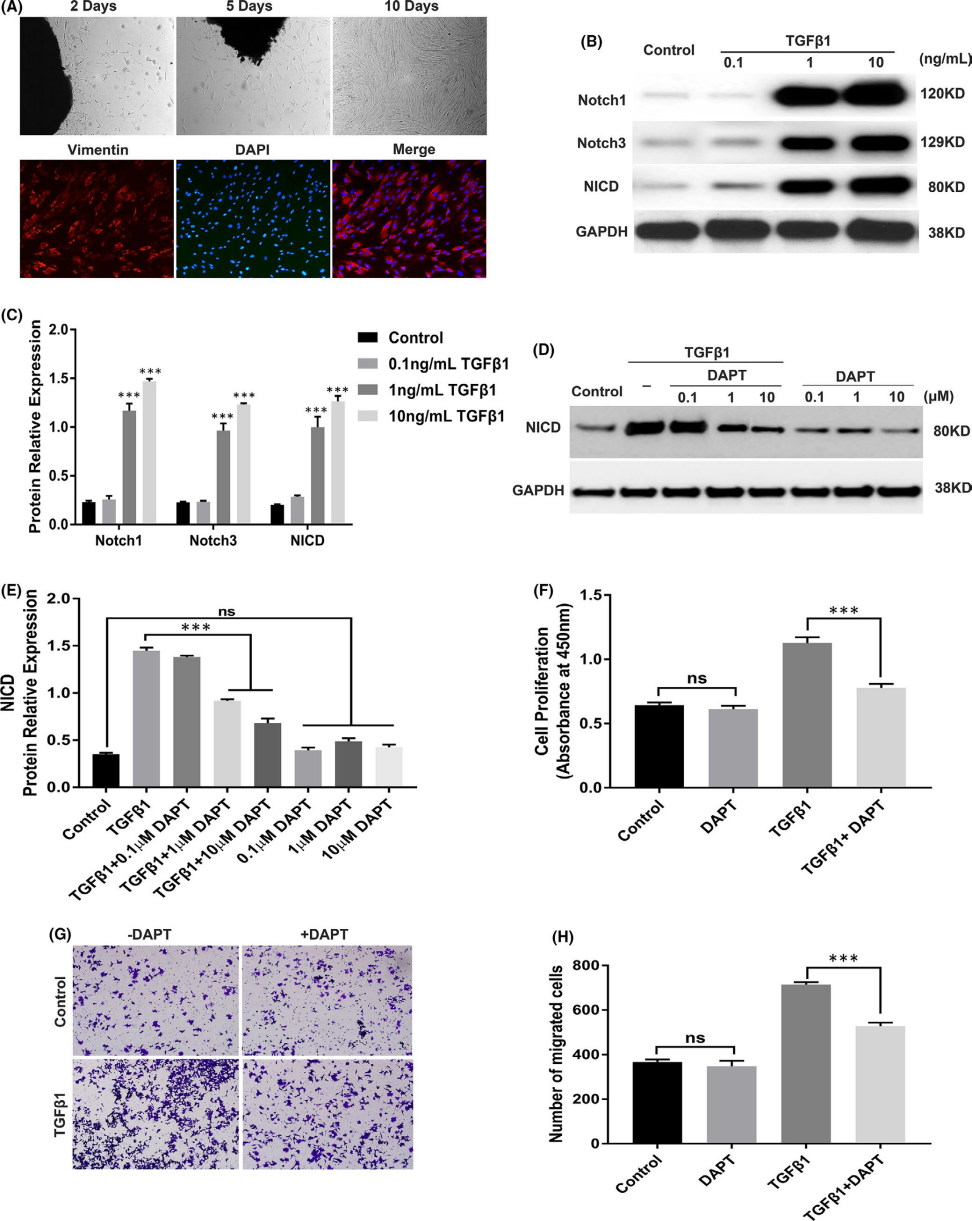
DAPT suppresses activation of Notch signalling, and the proliferation and migration of TGFβ1‐treated HUSFs. (A) Representative images of established primary HUSFs from fresh surgical specimens isolated from urethral scar tissues under a light microscope (20×) and after immunofluorescence staining of vimentin (red) under a fluorescent microscope (40×). (B) Western blot analysis of Notch receptors (Notch1, Notch3) and NICD expression in HUSFs stimulated with TGFβ1 (0.1, 1 or 10 ng/ml) for 24 h. (C) Quantification of the relative expression of Notch1, Notch3 and NICD proteins in TGFβ1‐treated HUSFs. (D) Western blot analysis of NICD expression in HUSFs pre‐treated with DAPT, with or without TGFβ1 (10 ng/ml) stimulation. The cells were pre‐treated with DAPT (0.1, 1 or 10 μM) for 24 h, after which they were stimulated with TGFβ1 (10 ng/ml) for 24 h or not further treated. (E) Quantification of the relative expression of NICD protein in HUSFs pre‐treated with DAPT, with or without TGFβ1 stimulation. (F) CCK‐8 assay of cell proliferation. HUSFs were pre‐treated with or without 10 μM DAPT for 24 h, after which they were stimulated with TGFβ1 (10 ng/ml) for 24 h or not further treated. (G) Cell migration assay. The images shown are representative of the migrated cells (40×). Pre‐treated HUSFs with or without DAPT treatment (10 μM for 24 h) were seeded in the upper chambers, after which they were stimulated with TGFβ1 (10 ng/ml) for 24 h or not further treated. (H) Quantification of migrated cells in TGFβ1‐treated HUSFs with or without DAPT treatment. Data are expressed as mean ± SEM (*n* = 3). * indicates *p* < 0.05, ** indicates *p* < 0.01, and *** indicates *p* < 0.001

Human urethral scar fibroblast cultures were successfully established from fresh surgical specimens. Typically, spindle‐shaped cells were observed within 2–5 days and reached 90%–100% confluence after approximately 10 days (Figure 2A). Intensive staining for vimentin was observed in the HUSFs, as analysed by immunofluorescence to identify fibroblasts (Figure 2A).

Human urethral scar fibroblasts were stimulated with different doses of TGFβ1 to investigate whether increased activation of the Notch pathway persists in TGFβ1‐treated HUSFs. Significant overexpression of Notch1, Notch3 and NCID was observed in TGFβ1‐treated HUSFs in a dose‐dependent manner (Figure 2B,C). Furthermore, to assess the potential role of Notch inhibitors in TGFβ1‐treated HUSFs, HUSFs were pre‐treated with DAPT and then stimulated with TGFβ1. A significant reduction in NICD expression occurred in the DAPT‐treated cells with TGFβ1 stimulation compared to the TGFβ1 group without DAPT treatment. However, NICD expression was barely affected in the DAPT‐treated cells without TGFβ1 stimulation compared to the observation in the control cells (Figure 2D,E).

We also assessed the effect of DAPT on the proliferation and migration of TGFβ1‐treated HUSFs. Inhibition of cell proliferation after the treatment with DAPT and TGFβ1 was confirmed by the reduced absorbance observed in the CCK‐8 assay (Figure 2F). Cell migration was clearly promoted by TGFβ1 but markedly inhibited by DAPT (Figure 2G,H). In addition, DAPT did not have any effects on the proliferation or migration of the DAPT‐treated cells without TGFβ1 stimulation. These data indicate that the increased activation of Notch signalling in HUSFs persists in vitro and that Notch inhibitors may play a vital role in the proliferation and migration of TGFβ1‐treated HUSFs.

### 3.3 DAPT inhibits ECM production, myofibroblast activation and the TGFβ signalling pathway in TGFβ1‐treated HUSFs

**FIGURE 3:**
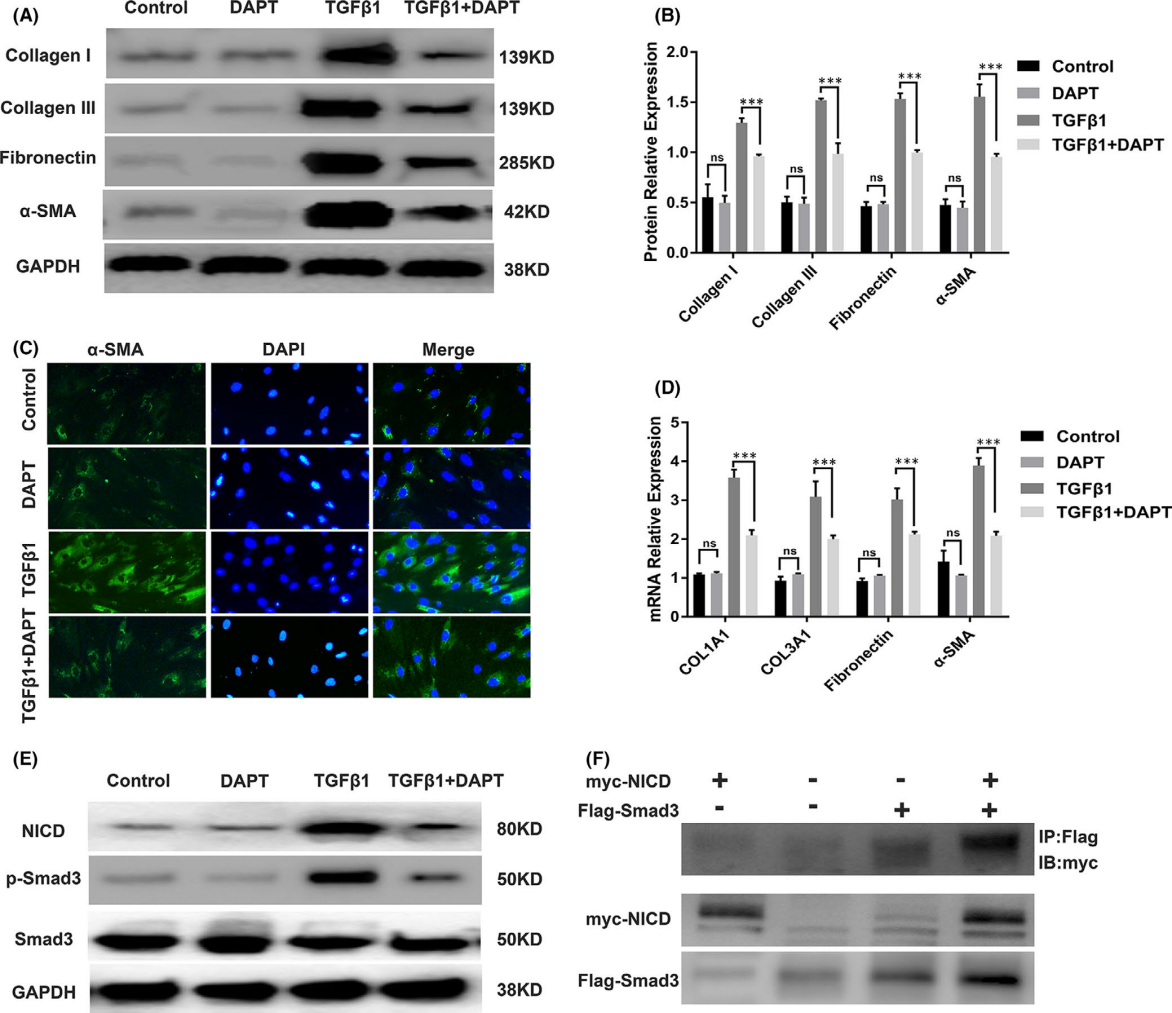
DAPT inhibits ECM production, myofibroblast activation and the TGFβ signalling pathway in TGFβ1‐treated HUSFs. (A) Western blot analysis of collagen I, collagen III, fibronectin and α‐SMA protein expression in HUSFs. The cells were pre‐treated with or without 10 μM DAPT for 24 h followed by stimulation with TGFβ1 (10 ng/ml) for 24 h. (B) Quantification of the relative expression of collagen I, collagen III, fibronectin and α‐SMA proteins in TGFβ1‐treated HUSFs with or without DAPT treatment. (C) Representative images showing immunofluorescence staining of α‐SMA (green) in TGFβ1‐stimulated HUSFs with or without DAPT treatment under a fluorescent microscope (40×). The cells were treated as indicated above. (D) Real‐time PCR analysis of the relative expression of COL1A1, COL3A1, fibronectin and α‐SMA mRNA in TGFβ1‐stimulated HUSFs with or without DAPT treatment. The cells were treated as indicated above. (E) Western blot analysis of NICD and phosphorylated Smad3 protein expression in TGFβ1‐stimulated HUSFs with or without DAPT treatment. The cells were treated as indicated above. (F) Coimmunoprecipitation of myc‐NICD and Flag‐Smad3. The anti‐Flag antibody directed against Flag‐tagged Smad3 pulls down myc‐tagged NICD only in Flag‐tagged Smad3 constructs. Data are expressed as mean ± SEM (*n* = 3). * indicates *p* < 0.05, ** indicates *p* < 0.01, and *** indicates *p* < 0.001

To evaluate the effects of DAPT on ECM production and myofibroblast activation, we incubated TGFβ1‐treated HUSFs with DAPT at an effective concentration based on the previous results (Figure 2D,F). Overexpression of ECM components and overactivation of myofibroblasts play a critical role in the pathogenesis of urethral fibrosis. Western blot analysis showed intense bands for collagen I, collagen III, fibronectin and α‐SMA following the TGFβ1 treatment; however, these were effectively attenuated by DAPT (Figure 3A,B). Intensive staining for α‐SMA protein was observed in TGFβ1‐stimulated HUSFs; however, fewer positive cells were observed after the DAPT treatment in the immunofluorescence analysis (Figure 3C). The levels of collagen type I alpha 1 chain (COL1A1), COL3A1, fibronectin and α‐SMA mRNA in the cells were upregulated following stimulation with TGFβ1 but downregulated by DAPT (Figure 3D). Interestingly, DAPT had no effect on the protein and mRNA expression levels in the cells that were not treated with TGFβ1. Together, these data suggest that stimulation of HUSFs with TGFβ1 results in the production of ECM and active myofibroblasts; however, these effects are reversed by DAPT in TGFβ1‐stimulated HUSFs.

It is reported that there is crosstalk between the Notch and TGFβ signalling pathway and that TGFβ/Smad is involved in the development of urethral fibrosis. Therefore, we explored whether the DAPT treatment affected this crosstalk in the HUSFs. As shown in Figure 3E, the expression of NICD and phosphorylated Smad3 was significantly higher in TGFβ1‐stimulated HUSFs than in the control cells. In contrast, NICD expression and phosphorylation of Smad3 were markedly decreased in the cells after the treatment with DAPT, indicating that suppression of Notch signalling results in inhibition of TGFβ signalling in HUSFs. Furthermore, to assess interactions between NICD and Smad3, co‐immunoprecipitation of myc‐NICD and Flag‐Smad3 was performed. As shown in Figure 3F, the anti‐Flag antibody directed against Flag‐tagged Smad3 reduced the expression of myc‐tagged NICD only in cells that also had the Flag‐tagged Smad3 construct, demonstrating the formation of a NICD‐Smad3 complex in the intact cells. These data indicate that DAPT can influence the crosstalk between Notch and the TGFβ pathway by targeting the interaction between NICD and Smad3.

### 3.4 Activation of Notch signalling promotes ECM production and myofibroblast activation in TGFβ1‐treated HUSFs

**FIGURE 4:**
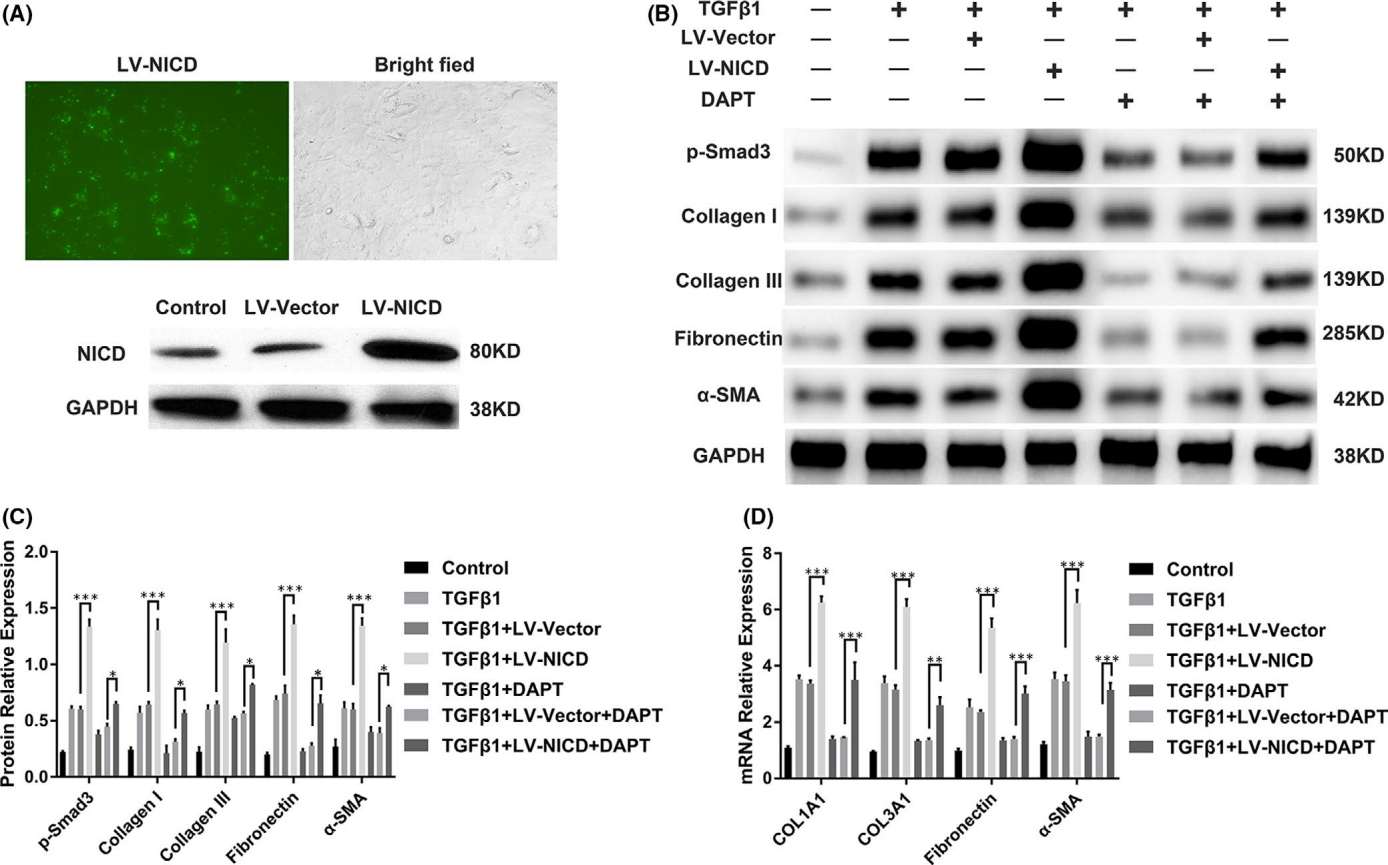
Activation of Notch signalling promotes ECM production and myofibroblast activation in TGFβ1‐stimulated HUSFs. (A) Representative immunofluorescence images of lentivirus overexpressing NICD (green) in HUSFs under a fluorescent microscope (20×) and Western blot analysis of NICD protein expression in vector (LV‐Vector)‐ or NICD (LV‐NICD)‐transfected HUSFs. The cells were serum‐starved overnight and incubated for 24 h. (B) Western blot analysis of p‐Smad3, collagen I, collagen III, fibronectin and α‐SMA protein expression in TGFβ1‐treated LV‐Vector or LV‐NICD HUSFs with or without DAPT treatment. The transfected (LV‐Vector or LV‐NICD) or non‐transfected HUSFs were pre‐treated with or without 10 μM DAPT for 24 h followed by stimulation with TGFβ1 (10 ng/ml) for 24 h. (C) Quantification of the relative expression of p‐Smad3, collagen I, collagen III, fibronectin and α‐SMA proteins in TGFβ1‐stimulated HUSFs (LV‐Vector or LV‐NICD) with or without DAPT treatment. (D) Real‐time PCR analysis of COL1A1, COL3A1, fibronectin and α‐SMA mRNA relative expression in TGFβ1‐stimulated HUSFs (LV‐Vector or LV‐NICD) with or without DAPT treatment. The cells were treated as indicated above. Data are expressed as mean ± SEM (*n* = 3). * indicates *p* < 0.05, ** indicates *p* < 40.01, and *** indicates *p* < 0.001

We demonstrated that the effects of stimulating HUSFs with TGFβ1 could be suppressed by DAPT, which indicates that the process is regulated by a Notch‐dependent pathway. To further confirm the effect of Notch signalling, HUSFs were transfected with lentiviral constructs overexpressing NICD (LV‐NICD) to activate Notch signalling. As shown in Figure 4A, LV‐NICD displayed a high transfection efficiency in the HUSFs, which was further confirmed by western blotting. Next, we cultured LV‐NICD HUSFs treated with TGFβ1 to determine whether NICD overexpression contributes to aberrant production of ECM and activation of myofibroblasts. Western blot analysis showed a significant increase in p‐Smad3, collagen I, collagen III, fibronectin and α‐SMA protein levels in NICD‐transfected HUSFs upon TGFβ1 stimulation compared to vector‐transfected HUSFs (Figure 4B,C). Importantly, NICD overexpression resulted in partial reversal of the ability of DAPT to inhibit ECM production and myofibroblast activation; however, this was not observed in the TGFβ1 + LV‐Vector + DAPT and TGFβ1 + LV‐NICD + DAPT cells (Figure 4B,C). Similar effects were observed for the mRNA levels (Figure 4D). Taken together, these results demonstrate that Notch signalling is essential for regulating ECM production and myofibroblast activation, indicating that Notch signalling participates in the development of urethral fibrosis.

### 3.5 DAPT ameliorates TGFβ1‐induced urethral fibrosis in rats

**FIGURE 5:**
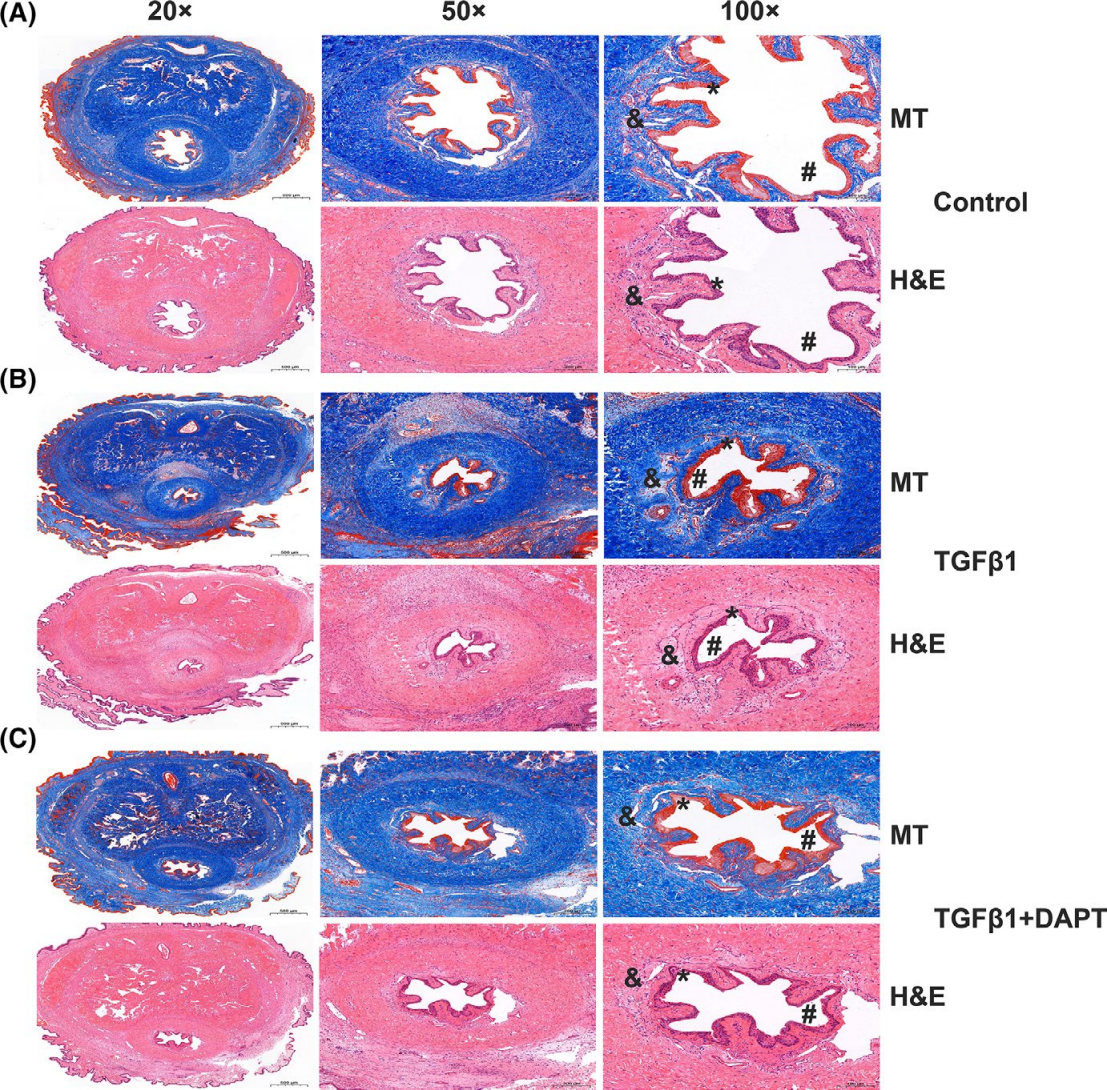
DAPT ameliorates TGFβ1‐induced urethral fibrosis in rats. Representative histologic assessment of MT‐ and H&E‐stained midshaft sections of rat penises at magnifications of 20×, 50×, and 100×. (A) MT staining (top) of a section from a sham control rat and H&E staining (bottom) on an adjacent section from the same rat. Normal urethra with intact stratified epithelium and normal distribution of collagen bundles beneath the basement membrane. (B) MT staining (top) of sections from a rat that was administered a local injection of TGFβ1 and H&E staining (bottom) of an adjacent section from the same rat. The image shows a narrow urethral lumen and irregularly arranged and dense collagen bundles beneath the urethral epithelium. (C) MT staining (top) of sections from a DAPT‐treated rat with urethral fibrosis and H&E staining (bottom) of an adjacent section from the same rat. The image shows mild submucosal urethral fibrosis and fewer collagen bundles deposited upon DAPT treatment beneath the urethral epithelium. Urethra lumen (#), urethral epithelium (*), urethral fibrosis (&)

H&E and MT staining were performed on the midshaft sections of rat penises to assess the effect of DAPT on TGFβ1‐induced urethral fibrosis. Comparative microscopic examination of representative H&E‐ and MT‐stained urethral sections revealed normal urethra with intact stratified epithelium and normal distribution of collagen bundles beneath the basement membrane in the control group (Figure 5A). In contrast, H&E‐ and MT‐stained sections from TGFβ1‐treated rats showed a narrow urethral lumen and irregularly arranged and dense collagen bundles beneath the urethral epithelium (Figure 5B). However, there was only mild submucosal urethral fibrosis and fewer collagen bundle depositions upon DAPT treatment, indicating that TGFβ1‐induced urethral fibrosis can be alleviated by DAPT (Figure 5C).

### 3.6 DAPT counteracts TGFβ1‐induced urethral fibrosis in rats by suppressing ECM production, myofibroblast activation and the TGFβ signalling pathway

**FIGURE 6:**
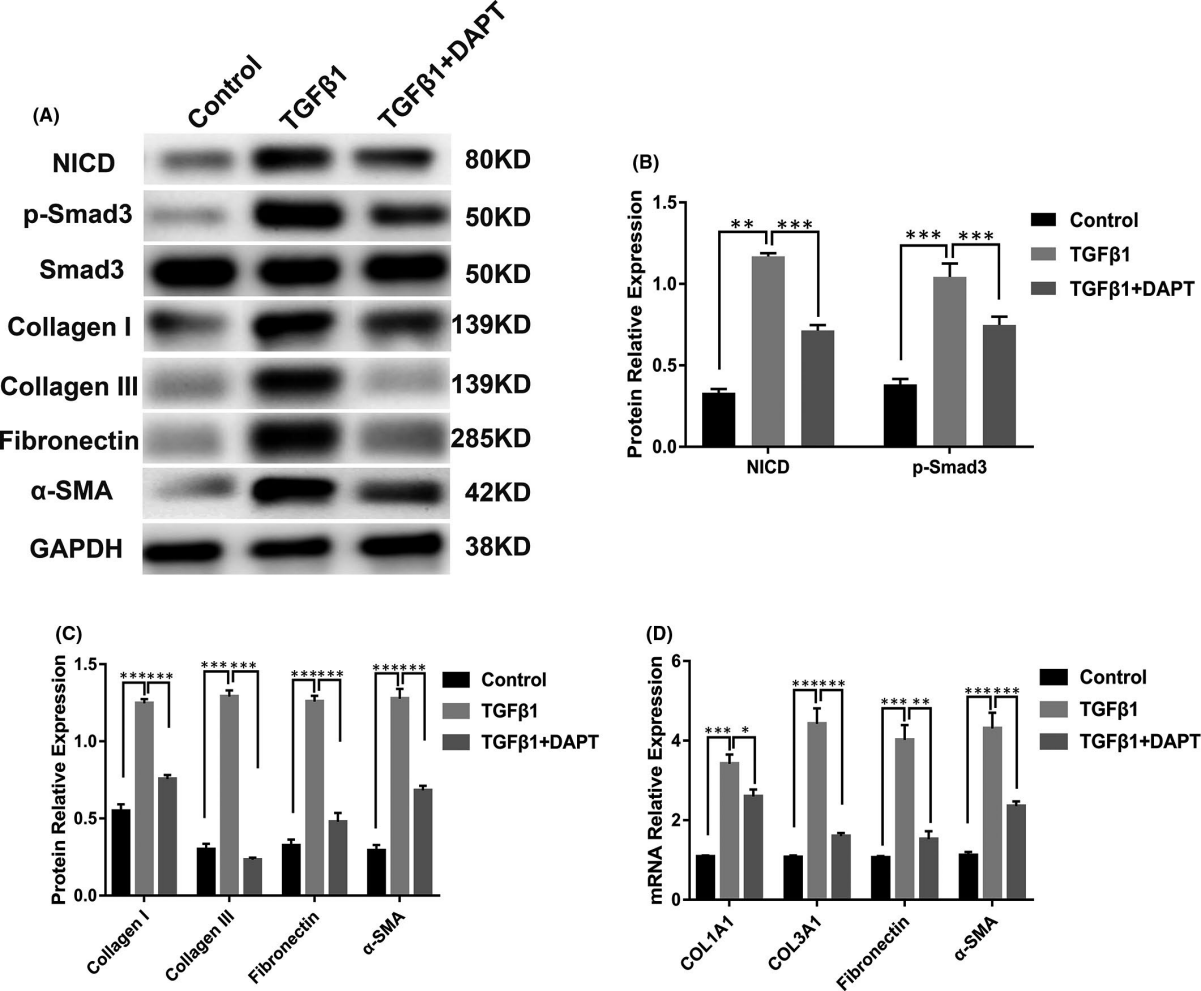
DAPT counteracts TGFβ1‐induced urethral fibrosis by suppressing ECM production, myofibroblast activation and the TGFβ signalling pathway. (A) Western blot analysis of NICD, p‐Smad3, collagen I, collagen III, fibronectin and α‐SMA protein expression in urethral tissues isolated from rats in the control, TGFβ1 and TGFβ1 + DAPT groups. (B) Quantification of the relative expression of NICD and p‐Smad3 proteins in urethral tissues isolated from rats in the control, TGFβ1 and TGFβ1 + DAPT groups. (C) Quantification of the relative expression of collagen I, collagen III, fibronectin and α‐SMA proteins in urethral tissues isolated from rats in the control, TGFβ1 and TGFβ1 + DAPT groups. (D) Real‐time PCR analysis of COL1A1, COL3A1, fibronectin and α‐SMA mRNA relative expression in urethral tissues isolated from rats in the control, TGFβ1 and TGFβ1 + DAPT groups. Data are expressed as mean ± SEM (*n* = 3). * indicates *p* < 0.05, ** indicates *p* < 0.01, and *** indicates *p* < 0.001

We investigated whether Notch signalling is overactivated in TGFβ1‐induced urethral fibrosis by analysing NICD expression by Western blotting. As shown in Figure 6A,B, the protein level of NICD was significantly higher in the TGFβ1 group than in the control group; however, this increased NICD expression was markedly alleviated after the DAPT treatment. After showing that DAPT inhibits the activation of TGFβ signalling in HUSFs, we tested whether DAPT suppresses this pathway in TGFβ1‐induced urethral fibrosis. As shown in Figure 6A,B, TGFβ1 induced a significant phosphorylation of Smad3; however, TGFβ1‐induced phosphorylation of Smad3 was partially inhibited by DAPT. These results indicated an interaction between NICD and Smad3 and suggests a potential mechanism by which Notch inhibitors could affect crosstalk between the Notch and TGFβ signalling pathways.

Furthermore, some pro‐fibrotic markers were analysed by Western blotting and real‐time PCR to further confirm whether DAPT ameliorates TGFβ1‐induced urethral fibrosis. The Western blot and real‐time PCR analyses showed significantly higher levels of collagen I, collagen III, fibronectin and α‐SMA in the TGFβ1 group than in the control group. Additionally, there was a significant decrease in collagen I, collagen III, fibronectin and α‐SMA expression in the DAPT‐treated group than in the TGFβ1 group (Figure 6A,C,D). These data corroborate the histologic findings.

## 4 DISCUSSION

In this study, we showed that Notch signalling is activated in the scar tissues of US patients with a prominent expression of Notch receptors (Notch1 and Notch3), Notch ligands (Jagged1, Jagged2, Delta3 and Delta4) and their target gene (Hes1). We further showed that the activation of Notch signalling upon stimulation with TGFβ1 has important implications for the activation of HUSFs. The Notch signalling inhibitor DAPT markedly suppressed the expression of collagen I, collagen III, fibronectin and α‐SMA, and the activation of TGFβ signalling, and finally inhibited TGFβ1‐induced urethral fibrosis.

It has been indicated that Notch signalling plays a vital role in the pathogenesis of fibrotic disorders.^20^ In pathological scars, impaired Notch signalling results in the alleviation of hypertrophic scar formation.^21^ Furthermore, it has been reported that Notch signalling is activated in patients with systemic sclerosis^12^ and in an animal model of kidney fibrosis.^22^ Therefore, Notch signalling has gained significance as a potential therapeutic target in fibrosis.

In the present study, we found that Notch signalling is activated in the scar tissues of patients with US. This important finding is a promising foundation based on which Notch signalling can be targeted to prevent the formation of US. Further, Notch ligand binding followed by γ‐secretase‐regulated cleavage is a crucial step in which Notch signalling activity can be pharmacologically mediated. Therefore, γ‐secretase inhibitors can suppress Notch signalling. Inhibition of Notch signalling using a γ‐secretase inhibitor has been reported to decrease the expression of Notch transcriptional target genes in cancer research.^23^

Crosstalk or interactions between TGFβ signalling and Notch signalling have been described. Recent studies have shown that Notch signalling can be induced under hypoxic conditions or by TGFβ.^24^ Moreover, recent work has indicated that the expression levels of Notch1 and Hes1 are increased in dermal fibroblasts following stimulation with TGFβ.^12^ In the present study, we first prepared HUSF cultures from fresh scar tissues isolated from US patients and continuously stimulated the cells with TGFβ1 to maintain the fibrotic properties of cells. Our results consistently demonstrated that Notch signalling is activated in HUSFs after stimulation with TGFβ1 and that this effect is suppressed by DAPT. These findings show that both TGFβ and Notch signalling may be involved in the development of urethral fibrosis. Furthermore, we showed that DAPT can inhibit the proliferation and migration of TGFβ1‐treated HUSFs. Notch signalling has been demonstrated to regulate the migration of fibroblasts.^25^ It has also been reported that pharmacological blockade of γ‐secretase could result in decreased proliferation of systemic sclerosis fibroblasts.^12^ Moreover, in a previous study, DAPT significantly decreased the proliferation of fibroblasts in pulmonary fibrosis.^26^ In this context, the current findings can explain why Notch inhibitors can inhibit urethral fibrosis by their effect on fibroblast function.

In this study, we established a rat model of TGFβ1‐induced urethral fibrosis, which is a well validated and described animal model,^19^, ^27^ for our investigations. One limitation of this pilot study was the use of an animal model of urethral fibrosis, as this model is not currently standardized. We believe that the local TGFβ1 injection we administered to induce urethral fibrosis in the animals is better than physical injury based on the generation of reproducible fibrosis. Owing to the small size of the animals, we were unable to evaluate the induced fibrosis by a direct method such as endoscopic or retrograde urethrography. However, the local TGFβ1 injection is a simple, inexpensive and reproducible technique for establishing the model. Changes in ECM components and differentiation of fibroblasts into myofibroblasts are the two critical processes involved in urethral fibrosis. Excessive deposition of ECM components, especially collagen, can lead to fibrosis. A previous report indicated that collagen I/III ratio changes in scar tissues in US.^4^ Furthermore, α‐SMA is a defining marker of myofibroblasts, which are essential cellular elements of fibrotic disease.^26^ Consequently, the number of myofibroblasts correlates with the severity of fibrosis.^28^ Our results showed that the Notch inhibitor DAPT downregulated the expression of collagen I, collagen III, fibronectin and α‐SMA in TGFβ1‐induced fibrosis in vitro and in vivo. Thus, the expression of these pro‐fibrotic genes may be regulated by Notch‐dependent signalling. Our findings also indicated that DAPT shows an antifibrotic effect in urethral fibrosis by inhibiting ECM production and fibroblast differentiation, which is consistent with a previous finding that Notch inhibitors have an antifibrotic effect.^29^

TGFβ signalling contributes to ECM production and fibroblast differentiation.^30^ However, whether Notch signalling activation in HUSFs regulates ECM production and differentiation remains unclear. Therefore, we transfected HUSFs with LV‐NICD and found that NICD overexpression resulted in a marked increase in ECM production and differentiation of HUSFs. Moreover, NICD overexpression resulted in a partial reversal of the antifibrotic effect of DAPT. These findings suggest that both activation of TGFβ signalling and Notch signalling are involved in urethral fibrosis. Our results also showed that DAPT suppressed activation of TGFβ signalling by downregulating the expression of phosphorylated Smad3, indicating that Notch signalling and TGFβ signalling could be integrated by direct protein‐protein interaction between NICD and Smad3. Recently, a Notch inhibitor was found to decrease Smad2 and Smad3 phosphorylation,^31^, ^32^ which indicates that Notch inhibitors may exert potent antifibrotic effects by inhibiting TGFβ signalling. In contrast, Notch signalling has also been reported to directly upregulate COL1A1 and COL1A2 promoter activity through a Hes1‐dependent mechanism in airway subepithelial fibrosis.^33^ Therefore, the precise mechanism underlying Notch downstream regulation of TGFβ/Smad signalling needs to be clarified in further experiments.

## 5 CONCLUSION

In conclusion, our results indicate that both Notch and TGFβ signalling contribute to urethral fibrosis and provide direct evidence that Notch signalling is activated in urethral fibrosis. Additionally, the Notch inhibitor DAPT significantly attenuates TGFβ1‐induced urethral fibrosis by suppressing ECM production and fibroblast differentiation. Our current findings may provide new promising targets for inhibiting the development of urethral fibrosis.

## CONFLICT OF INTEREST

The authors declare that they have no competing interests.

## AUTHOR CONTRIBUTIONS

**Shanlong Huang:** Conceptualization (lead); Data curation (lead); Formal analysis (lead); Investigation (lead); Methodology (lead); Writing‐original draft (lead). **Delai Fu:** Conceptualization (supporting); Data curation (supporting); Methodology (supporting); Supervision (supporting); Writing‐review & editing (supporting). **Ziyan Wan:** Data curation (supporting); Investigation (supporting); Methodology (supporting); Resources (supporting). **Min Li:** Conceptualization (supporting); Data curation (supporting); Methodology (supporting); Supervision (supporting); Writing‐original draft (supporting). **Hecheng Li:** Funding acquisition (supporting); Methodology (supporting); Project administration (supporting); Resources (supporting); Writing‐review & editing (supporting). **Tie Chong:** Funding acquisition (lead); Project administration (lead); Supervision (lead); Writing‐review & editing (lead).

## Data Availability

The data that support the findings of this study are available from the corresponding author upon reasonable request.
